# Effectiveness of Telemedicine in Controlling Hyperglycemia Among Diabetic Patients on Insulin Therapy in Primary Care: A Systematic Review and Meta-Analysis

**DOI:** 10.7759/cureus.50045

**Published:** 2023-12-06

**Authors:** Azhar Al Ibrahem, Asma M Al Omran, Duaa T Alaithan, Kawthar A Aldandan, Mariyyah A Al Shaghab, Abrar M Alkhudhayr, Basmah J AL Ramadhan, Salman J Alshehab, Mohammed N Alkhudhair, Ali A Alamer, ‏Ali M Aldrees

**Affiliations:** 1 Family Medicine, Al-Ahsa Health Cluster, Ministry of Health, Al-Ahsa, SAU

**Keywords:** primary care, insulin, diabetic patients, hyperglycemia, telemedicine

## Abstract

This study investigates the effectiveness of telemedicine in managing glucose levels in insulin-treated diabetes patients compared to standard care. Adhering to the Preferred Reporting Items for Systematic Reviews and Meta-Analysis (PRISMA) criteria and Cochrane's risk of bias tool, an analysis of five selected studies reveals telemedicine as a potent tool in diabetes management. Fasting blood sugar (FBS) test results from two studies involving an eight-hour fast with 109 participants demonstrate a significant superiority of telemedicine over usual care (Tau2 = 1.63; Chi2 = 1.01, df = 1, P = 0.32; Z = 2.43, P = 0.02), highlighting its potential in short-term blood sugar stabilization. Postprandial plasma glucose (PPBG) test outcomes suggest comparable efficacy in managing post-meal blood glucose levels with telemedicine. Additionally, analysis of glycated hemoglobin (HbA1c) levels across all five studies indicates telemedicine's equivalence to traditional care in maintaining HbA1c levels among insulin-treated patients, affirming its efficacy in primary care. While emphasizing telemedicine's effectiveness in managing FBS levels, a critical aspect of diabetes control, among patients utilizing insulin therapy in primary care, the study underscores the need for more extensive, large-scale research to fully comprehend its impact on diabetes management.

## Introduction and background

Diabetes mellitus (DM) is a severe, chronic metabolic condition necessitating lifelong monitoring. According to the International Diabetes Federation's 10th edition, an estimated 537 million adults worldwide are affected. This number is expected to reach 643 million by 2030 (11.3%), making it one of the greatest epidemics globally [1]. Over 90% of those who are affected have type 2 DM (T2D) [1]. The rise in the prevalence of T2D is linked to an aging population, urbanization, sedentary lifestyles, and the consumption of unhealthy diets. On the other hand, early detection and effective treatments have led to improved survival rates [2].

Individuals with diabetes must adhere to a strict management plan, including taking medication, lifestyle modifications in the form of a healthy diet, regular physical activity, and maintaining a healthy weight. Uncontrolled rises in blood glucose levels (defined as A1c ≥ 7%) can lead to irreversible damage and severe complications that negatively impact patients’ health and quality of life. Patient engagement and self-care play pivotal roles in diabetes management, significantly influencing glycemic control. Regular follow-up and consistent education are standard management practices, but they pose a challenge to patients in rural areas with limited access to healthcare [3].

Current diabetes guidelines recommend regular healthcare visits every three months to measure glycated hemoglobin (HbA1c), adjust therapy, screen for complications and comorbidities, and optimize overall health [4]. Telemedicine has grown rapidly and is defined by the American Telemedicine Association as the electronic exchange of medical information from one site to another via electronic communications to enhance patients' clinical health status. This technology has revolutionized healthcare and can effectively be complementary to regular office visits. Telemedicine utilizes various technologies, such as applications, wireless tools, and smartphones, to evaluate, diagnose, and treat patients in remote regions [5]. In comparison to telemedicine, telehealth delivers a wider range of digital health services and should complement in-person visits to enhance glycemic control in uncontrolled diabetes [6].

Research indicates that telehealth methods can significantly lower A1C levels in people with T2D [7]. Findings suggest telemedicine is effective in delivering healthcare to type 1 diabetes (T1D) patients by saving the patient's time and money, resulting in high appointment adherence rates and patient satisfaction [8]. The effect of telemedicine on HbA1C levels was most pronounced in studies where participants started with higher baseline HbA1C concentrations and underwent changes in their prescribed medications [9-10]. In addition, it aids in addressing and managing diabetes-related comorbidities like hypertension and dyslipidemia [11-12].

Previous meta-analyses found telemedicine interventions more effective than usual care in managing diabetes, especially in older patients and those with longer intervention durations [13]. Another meta-analysis focused on gestational diabetes mellitus (GDM), showing significant improvements in controlling HbA1c and reducing the risk of maternal and neonatal complications in telemedicine groups [14].

Despite these advantages, several barriers hinder telemedicine acceptance among diabetic patients, including lack of technical knowledge, absence of standards, doubts about efficacy, and altered practitioner-patient interactions [15].

The objective of this study is to assess the effectiveness of a telemedicine system in improving glucose control among patients receiving insulin treatment for T1D, T2D, and GDM in comparison to the current standard of care.

## Review

Methods of systematic review

Literature Search Strategy

The Preferred Reporting Items for Systematic Reviews and Meta-Analysis (PRISMA) criteria were followed for conducting this systematic review [16]. The International Prospective Register of Systematic Reviews (PROSPERO) has the study protocol listed under ID# CRD42023461949 [17]. A thorough electronic search was carried out in the databases PubMed, Cochrane Library, Medical Literature Analysis and Retrieval System Online (MEDLINE), and Excerpta Medica database (Embase) in September 2023. To find relevant papers, Medical Subject Heading (MeSH) terms and associated keywords were combined. The keywords used in the search were "Telemedicine" AND "Hyperglycemia" AND ("Diabetic Patients") AND ("Insulin Therapy" OR "Insulin Treatment") AND ("Primary Care" OR "Primary Healthcare") AND "RCT". Studies were included in the search without regard to when they were published.

Inclusion/Exclusion Criteria

This review focused on randomized controlled trials (RCTs) utilizing both crossover and parallel designs. The study participants were adults aged 18 and above who were prescribed insulin for T1D, T2D, or GDM. No language restrictions were applied. Excluded from the review were non-randomized trials, studies that did not report relevant outcomes for the clinical inquiries, research involving children or individuals below 18 years, investigations not using insulin as a diabetes treatment, and duplicate studies.

Study Selection

To ease the elimination of duplicate entries, all records obtained from the initial search were imported to Mendeley (Elsevier Limited, London, UK). The deduplicated results were then imported into Rayyan (Rayyan Systems Inc., Cambridge, MA, and put through a preliminary relevance check based on the details in the titles and abstracts. Two authors carried out the screening procedure to ascertain which studies satisfied the predetermined inclusion and exclusion criteria. Following the initial screening, the whole texts of the papers that made it beyond the first step were collected and carefully reviewed by three authors, Al Ibrahem A., Al Omran A. M., and Alaithan D. T., who ultimately decided whether to include or exclude each study.

Data Extraction and Analysis

Study identifications, such as author(s), year of publication, country of origin, study design, sample size, follow-up period, and inclusion/exclusion criteria, were included in the data retrieved from the preserved studies. In addition, participant data like age and sex distribution, diabetes type, and medication use were noted. The different telemedicine surface types were covered by the interventional characteristics. Diabetes-related patient outcomes (physiological, psychological, or behavioral) fell under the area of outcome measurements. Glycemic control by fasting blood sugar (FBS), postprandial plasma glucose (PPBG), and the change in glycated hemoglobin (%) is the main result for T1D, T2D, and GDM. The study's main conclusions, any limitations that the authors acknowledged, and any suggestions or implications for future research were all included in the study's results section.

Quality of the Included Studies and the Risk of Bias Assessment

For RCTs, we made use of Cochrane's risk of bias tool. Studies that posed a severe randomized bias risk were not included. This will entail assessing each study in light of several criteria and categorizing them as having a low, high, or uncertain risk of bias for each.

Statistical Analysis

All analyses were conducted using RevMan version 5.4.1 (Review Manager, (Computer program), The Cochrane Collaboration, 2020). We extracted the means and standard deviations of the scores for the questions evaluating the improvements of FBS, PPBS, and HbA1C from the included studies, both in the telemedicine and control groups. A weighted mean difference with 95% confidence intervals (CIs) was pooled using a fixed-effects model. Forest plots were created to evaluate the results of pooling. A p-value less than 0.05 was considered significant. Heterogeneity between trials was assessed using the Higgins I2 test according to the Cochrane Handbook.

Results

Study Description

**Figure 1:**
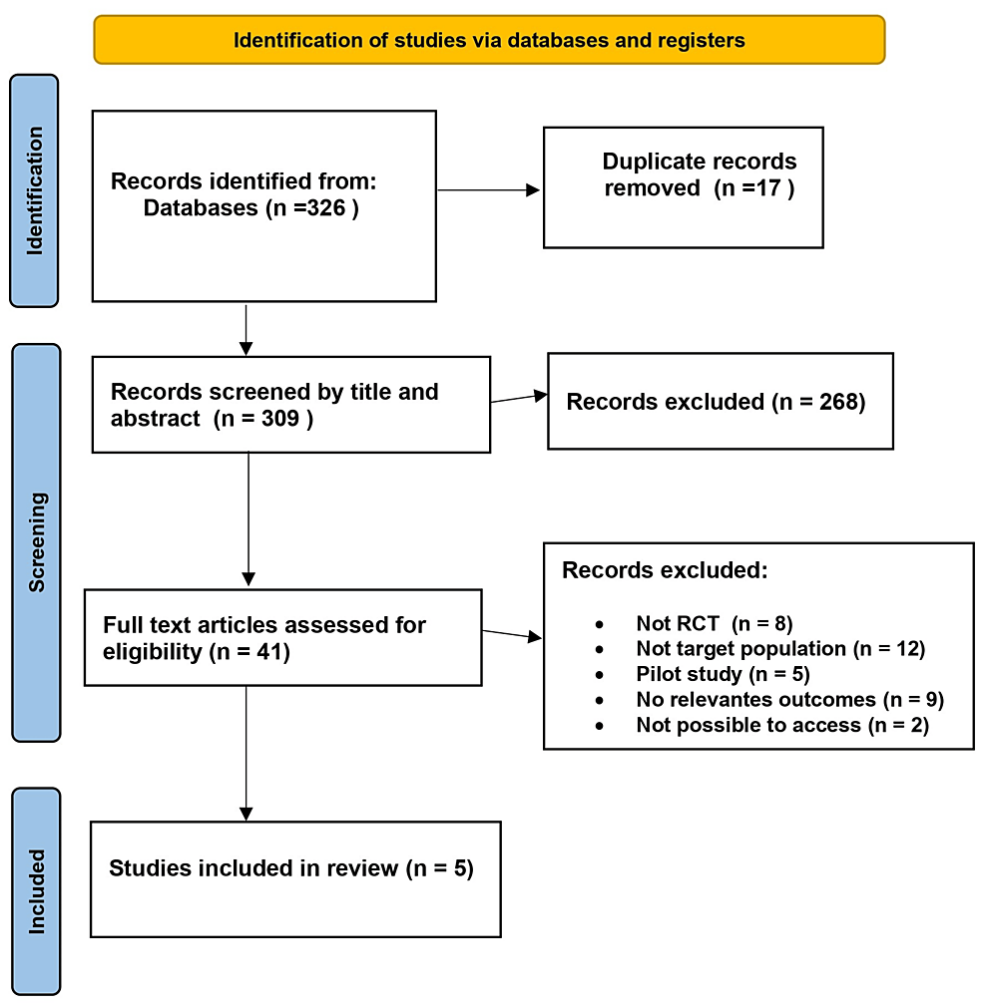
A PRISMA flow diagram depicting the selection of studies PRISMA: Preferred Reporting Items For Systematic Review and Meta-Analysis; RCT: randomized control trial

The initial search of the database identified 326 relevant studies meeting our inclusion criteria. After eliminating 17 duplicate studies, 309 articles were screened based on title and abstract. Among these, 268 studies were excluded for not meeting our criteria. An additional 36 studies that were subjected to full-text assessment were excluded due to other reasons such as irrelevant participants, incomplete outcome variables, or insufficient data for further analysis. Finally, five studies aligning perfectly with our criteria were included. The methodological quality of these chosen studies was evaluated using the PRISMA protocol (Figure 1).

The five selected studies were conducted in distinct countries: Italy, Poland, France, Sweden, and Spain. Regarding the study participants, two studies included both T1D and T2D patients, while another study focused exclusively on individuals with T2D. Additionally, one study targeted adults diagnosed with T1D, and the fifth study investigated T2D during pregnancy and the postpartum period. All these studies used HbA1c (with some also incorporating FBS and PPBG) as primary outcome measures, hypoglycemia events, and quality of life as secondary parameters. A summary of the key data is Table 1.

**Table 1:** Characteristics of studies included in the review TM: telemedicine; TH: telehealth; HbA1c: glycated hemoglobin; DIABEO: system combining mobile app software for telemonitoring

Study	Participants	Sex, Male (N)	Sex, Female (N)	Country	Intervention vs. Control	Duration	Results	Conclusions
Di Molfetta et al., 2022 [18]	123 patients	67	56	Italy	Glucoonline TM system vs. regular glucose meter	6 months	Group A had a significant reduction in HbA1c (-0.38%, p<0.05) after six months.	The Glucoonline TM system improved metabolic control. Telemedicine services have the potential to support diabetes self-management.
Fedak et al., 2011 [19]	95 patients	51	44	Poland	Telehome monitoring vs. medical consultations and home visit	6 months	Overall reduction in HbA1c in both groups after six months. Significant difference observed in non-insulin-requiring patients.	Telehome monitoring is effective in controlling type 2 diabetes in primary care, especially for certain patient profiles.
Franc et al., 2020 [20]	665 patients	323	342	France	DIABEO vs. standard care	12 months	DIABEO users showed a significant reduction in HbA1c compared to standard care after 12 months.	DIABEO usage resulted in a clinically and statistically significant reduction in HbA1c levels.
Linden et al., 2017 [21]	158 patients	0	158	Sweden	Web-based support vs. standard care	From early pregnancy till six months after childbirth	No differences were found in general well-being or self-efficacy of diabetes management between groups.	Web-based support did not show superiority over standard care in postpartum diabetes management. Further intervention studies are needed.
Ballesta et al., 2023 [22]	55 patients	27	28	Spain	TH vs. in-person visits	6 months	No significant differences in HbA1c between groups. The TH group showed improvements in glucose metrics and quality of life.	Telehealth is comparable to in-person visits for HbA1c levels with improvements in some aspects of health-related quality of life. More efficient timing of TH visits needs further evaluation.

Risk of Bias

The risk of bias assessment for the included studies revealed that all studies had a low risk of bias in the generation of random sequences. However, one study had an unclear bias risk due to the absence of classification for allocation concealment, specifically related to selection bias.

Given that telemedicine was the consistent intervention in all studies and participants were generally unblinded due to the nature of the intervention, a high risk of bias was identified in terms of blinding participants (performance bias) across most studies. There was one exception where participant awareness of the study was not clearly defined, leading to an uncertain bias risk in this specific case.

The risk of detection bias was unclear in three studies due to insufficient information, while two studies were classified as high-risk due to identifiable detection bias. However, factors related to attrition, reporting, and other biases were evaluated as low-risk across all studies.

Table 2 presents a detailed visual representation of the risk of bias analysis.

**Table 2:** Summary of risk of bias assessment Risk of bias legend: (A) random sequence generation (selection bias); (B) allocation concealment (selection bias); (C) blinding of participants and personnel (performance bias); (D) blinding of outcome assessment (detection bias); (E) incomplete outcome data (attrition bias); (F) selective reporting (reporting bias); (G) other biases.

Study	Risk of Bias						
	A	B	C	D	E	F	G
Ballesta et al., 2023 [22]	Low	Low	High	High	Low	Low	Low
Di Molfetta et al., 2022 [18]	Low	Low	Unclear	Unclear	Low	Low	Low
Fedak et al., 2011 [19]	Low	Unclear	High	Unclear	Low	Low	Low
France et al., 2020 [20]	Low	Low	High	Unclear	Low	Low	Low
Linden et al., 2017 [21]	Low	Low	High	High	Low	Low	Low

Outcome Measures

In our research, we specifically chose key blood glucose tests commonly utilized in diabetes studies due to their clinical importance in evaluating diabetes management. The FBS levels, measured after an eight-hour fast, are vital for understanding immediate blood sugar fluctuations and serve as a standard diagnostic criterion for diabetes. The PPBG levels indicate blood glucose levels after meals, offering insights into the body's ability to handle glucose intake. Additionally, we assessed the HbA1c levels, which reflect average blood sugar levels over several months and are widely regarded as a benchmark in long-term diabetes care. Our focus on these specific tests was to comprehensively evaluate telemedicine's impact on diverse aspects of diabetes control, covering both short-term fluctuations and long-term glycemic stability.

Among the five studies meeting our selection criteria, one study stood out for its unique approach, incorporating three intervention groups: arm 1 (standard care), arm 2 (mobile app alone), and arm 3 (mobile app + telemonitoring by trained nurses). Given that most intervention groups employed the same outcome measures, we initially focused on FBS.

**Figure 2:**
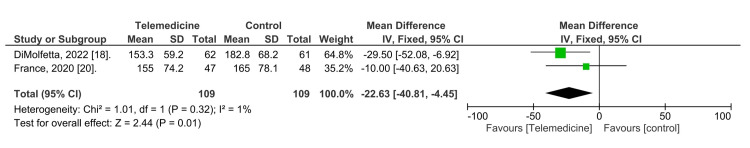
A meta-analysis comparing the effect of telemedicine and a control group on fasting blood sugar control.

The FBS study involved a total of 109 participants across both telehealth intervention and control groups from two separate studies. The analysis revealed minimal heterogeneity (Tau2 = 1.63; Chi2 = 1.01, df = 1, P = 0.32) and a significant overall effect (Z = 2.43, P = 0.02), indicating the superiority of telemedicine over usual care groups in this context, as illustrated in the forest plot in Figure 2.

**Figure 3:**
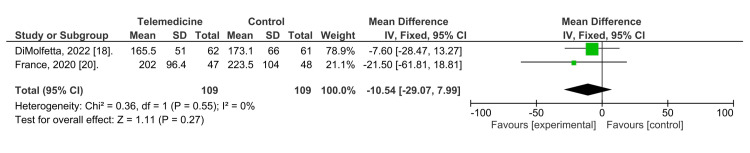
A meta-analysis comparing the effect of telemedicine and a control group on postprandial plasma glucose control.

Furthermore, our analysis focused on short-term changes in blood glucose levels, measured by the PPBG test, and was conducted in two studies with a total of 109 subjects in both telehealth intervention and control groups. The analysis showed no significant heterogeneity (Tau2 = 0.00; Chi2 = 0.36, df = 1, P = 0.55) and an insignificant overall effect (Z = 1.11, P = 0.27). In this analysis, no significant differences were observed between telemedicine and usual care groups, as shown in Figure 3.

**Figure 4:**
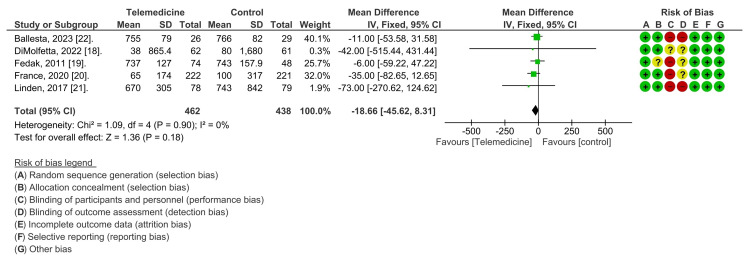
A meta-analysis comparing the effect of telemedicine and a control group on hemoglobin A1c levels.

Additionally, our analysis concentrated on HbA1c as an indicator to assess the impact of telemedicine on DM control. All five studies were included in this analysis. Upon conducting a thorough analysis, our study compared telemedicine intervention groups, consisting of 657 participants, with control groups comprising 438 individuals. The statistical examination displayed no significant heterogeneity (Chi2 = 1.13, df = 4, P = 0.89), with an I2 statistic of 0%, indicating uniformity among the studies. The overall effect was deemed insignificant (Z = 1.39, P = 0.17). indicating that there was no substantial difference in HbA1c levels between the control and intervention groups, as shown in the forest plot in Figure 4.

This highlights that telemedicine is as effective as usual care in controlling HbA1c levels among patients receiving insulin therapy, thus affirming its efficacy in primary care settings.

In summary, our findings emphasize telemedicine's effectiveness in managing FBS levels, a vital aspect of diabetes control among diabetic patients using insulin therapy in primary care settings. These findings underscore the significant impact telemedicine can have on improving glycemic control in this patient population.

Discussion

In our systematic review and meta-analysis, we aimed to evaluate the effectiveness of telemedicine in managing blood sugar levels among insulin-dependent patients, encompassing those with T1D, T2D, and GDM. The results of our study underscore telemedicine's promise within standard healthcare settings. Specifically, telemedicine demonstrated superior control of blood glucose levels, particularly in fasting blood glucose (FBG), compared to traditional methods. Moreover, our analysis indicated that telemedicine was as effective as traditional approaches when assessing PPBG and HbA1C levels. These findings represent a significant advancement in diabetes care and accentuate telemedicine's potential to innovate insulin-dependent diabetes management across diverse patient populations.

Comparing our findings with existing literature, numerous studies have elucidated the benefits of telemedicine in diabetes management, especially for insulin-dependent patients. Tchero et al. (2019) conducted a comprehensive meta-analysis of 42 RCTs, focusing on telemedicine's clinical effectiveness in diabetes. Their study, involving 6,170 participants, revealed that telemedicine interventions outperformed usual care in managing diabetes. Notably, older patients and those exposed to longer intervention durations experienced the most significant benefits, especially in reducing HbA1C levels among individuals with T2D [13]. Similarly, Polisena et al. (2009) demonstrated the effectiveness of telehealth interventions, including telemonitoring and tele-education, in improving clinical outcomes and reducing healthcare service utilization in diabetic patients [23-27]. Additionally, Shea et al. (2016) found that a telemedicine intervention for diabetes management improved glycemic control as effectively as traditional face-to-face methods [28, 29]. These studies, coupled with our findings, underscore telemedicine's potential to enhance care for insulin-dependent diabetic patients.

Furthermore, a meta-analysis conducted by Weihua Xie et al. in 2020 focused on the effectiveness of telemedicine interventions for GDM. When comparing the telemedicine group to standard care, the analysis of data from 32 RCTs with 5,108 patients showed notable improvements in controlling HbA1C, fasting blood, and two-hour PPBG levels [14]. Another study by Shalom et al. (2021) introduced a distributed decision support system that provided personalized, evidence-based guidance through mobile devices, particularly benefiting GDM patients [30,31]. This study highlighted telemedicine's potential for offering personalized care to insulin-dependent diabetic patients.

The absence of data concerning patient satisfaction, quality of life, psychiatric aspects, and cost-effectiveness in our study stems from these variables not being addressed in the research results we examined. Among the studies included, only Fedak et al. (2011) integrated certain psychological aspects into their findings. Specifically, in the control group, 27% of participants reported feelings of depression, compared to 19% in the telemedicine group. Similarly, 52% of individuals in the control group experienced anxiety and fear, whereas this percentage decreased to 35% in the telemedicine group. Additionally, a sense of inability to control one's life was reported by 10% of the control group participants, compared with 6% in the telemedicine group [21]. Another study conducted by Ballesta et al. (2023) thoroughly examined cost-effectiveness and patient satisfaction. The research revealed significantly lower total costs for participants in the telehealth group [22, 32]. Moreover, participants in the age group of 12 to 48 years expressed high levels of satisfaction and comfort with the virtual clinic, especially in cases of T1D, compared to traditional in-person visits [22].

These disparities in psychological well-being between the control and telemedicine groups, as revealed in Fedak et al.’s study, emphasize the potential impact of telemedicine interventions on patients’ mental health. The reduced prevalence of depressive feelings, anxiety, and perceived lack of control in the telemedicine group underscores the positive influence of telemedicine on the psychological aspects of the patient experience. The findings from Ballesta et al.'s study highlight the economic benefits of telehealth interventions, demonstrating lower costs for participants and a higher level of patient satisfaction, particularly among younger individuals. These results further support the efficacy and acceptability of telemedicine in healthcare contexts, emphasizing its potential to provide cost-effective and satisfying healthcare experiences, especially for specific demographic groups.

However, our study has some limitations that warrant consideration. A notable constraint is the limited scope of the included trials, characterized by small sample sizes and relatively short durations. This limitation is a result of challenges in resource availability and the difficulty in recruiting a larger cohort of participants with insulin-treated diabetes. Given the nature of insulin-treated diabetes, a deliberate decision was made to opt for a smaller, more manageable sample size, allowing for a thorough investigation within the constraints of available resources. Additionally, our study lacks a comprehensive cost-benefit analysis, particularly in terms of the economic aspects of telemedicine interventions. A thorough examination of the financial implications of implementing telemedicine for insulin-treated diabetes is essential for informing healthcare decision-makers. Furthermore, the observed variability in telemedicine technologies, the care provided to control groups, and the demographic characteristics of the studied populations may introduce potential inconsistencies in our results. Recognizing that these variations may have influenced the outcomes of our study is crucial for a nuanced interpretation of the findings.

Given our findings and the identified limitations, we recommend that future research focus on standardizing telemedicine interventions and assessing their long-term impact on insulin-dependent diabetic patients. Standardization is pivotal to ensuring consistent and reliable outcomes across diverse patient populations. Additionally, detailed assessments of telemedicine's long-term effects on glycemic control, patient satisfaction, and overall quality of life are crucial. Moreover, investigating the economic implications of widespread telemedicine adoption, including its cost-effectiveness and potential savings for both healthcare systems and patients, is essential. Understanding the socioeconomic impact of telemedicine can inform policy decisions and facilitate its seamless integration into healthcare practices. By addressing these aspects, future research can significantly contribute to advancing telemedicine's role in managing insulin-dependent diabetes, ultimately enhancing patient outcomes and transforming the landscape of diabetes care.

## Conclusions

In conclusion, this systematic review and meta-analysis have provided valuable insights into the effectiveness of telemedicine in managing blood glucose levels among insulin-dependent diabetic patients, including those with T1D, T2D, and GDM. Our findings indicate that telemedicine, particularly in the context of primary care settings, offers significant advantages, especially in controlling FBS levels, a critical aspect of diabetes management. While demonstrating comparable efficacy to traditional methods in impacting PPBG and HbA1c levels, telemedicine continues to advance toward more personalized, cost-effective, and accessible care.

However, it is essential to acknowledge the limitations of the existing studies, such as small sample sizes and short durations, which point to the need for more extensive and standardized research. Future investigations should focus on standardizing telemedicine interventions, assessing long-term effects on glycemic control and overall quality of life, and comprehensively evaluating their economic implications.
